# TDP-43 as a potential retinal biomarker for neurodegenerative diseases

**DOI:** 10.3389/fnins.2025.1533045

**Published:** 2025-02-12

**Authors:** Margit Glashutter, Printha Wijesinghe, Joanne A. Matsubara

**Affiliations:** ^1^Department of Ophthalmology and Visual Sciences, Faculty of Medicine, Eye Care Centre, University of British Columbia, Vancouver, BC, Canada; ^2^Faculty of Medicine, University of British Columbia, Vancouver, BC, Canada; ^3^Djavad Mowafaghian Centre for Brain Health, The University of British Columbia, Vancouver, BC, Canada

**Keywords:** TDP-43 proteinopathy, neurodegenerative disease, biomarker, retina, eye, brain

## Abstract

TDP-43 proteinopathies are a spectrum of neurodegenerative diseases (NDDs) characterized by the pathological cytoplasmic aggregation of the TDP-43 protein. These include amyotrophic lateral sclerosis (ALS), frontotemporal lobar degeneration (FTLD), Alzheimer’s disease (AD), chronic traumatic encephalopathy (CTE), and others. TDP-43 in the eye shows promise as a biomarker for these NDDs. Several studies have identified cytoplasmic TDP-43 inclusions in retinal layers of donors with ALS, FTLD, AD, CTE, and other conditions using immunohistochemistry. Our findings suggest that pathological aggregates of TDP-43 in the human retina are most prevalent in FTLD-TDP, ALS, and CTE, suggesting these diseases may provide the most reliable context for studying the potential of TDP-43 as a retinal biomarker. Animal model studies have been pivotal in exploring TDP-43’s roles in the retina, including its nuclear and cytoplasmic localization, RNA binding properties, and interactions with other proteins. Despite these advances, more research is needed to develop therapeutic strategies. A major limitation of human autopsy studies is the lack of corresponding brain pathology assessments to confirm TDP-43 proteinopathy diagnosis and staging. Other limitations include small sample sizes, lack of antemortem eye pathology and clinical histories, and limited comparisons across multiple NDDs. Future directions for the TDP-43 as a retinal biomarker for NDDs include retinal tracers, hyperspectral imaging, oculomics, and machine learning development.

## 1 Introduction

TAR DNA binding protein of 43 kDa (TDP-43) is a highly conserved protein coded by the *TARDBP* gene and is expressed ubiquitously (de Boer et al., 2021). Its primary functions include RNA/DNA homeostasis, protein quality assurance, mitochondrial autophagy, axonal transport, and stress granule formation (Liao et al., 2022; Babazadeh et al., 2023). Further, it appears to be critical in early embryogenesis for the formation of central neuronal cells (de Boer et al., 2021). Despite being a predominantly nuclear protein, TDP-43 can shuttle between the nucleus and cytoplasm via nuclear localization and export signals, with up to ∼30% of it found in the cytoplasm (Ayala et al., 2008; Scotter et al., 2015; Liao et al., 2022). TDP-43 is primarily involved in mRNA splicing and microRNA modulation for numerous transcripts, making its own homeostasis essential for proper cellular function (Scotter et al., 2015; Tamaki and Urushitani, 2022).

**FIGURE 1:**
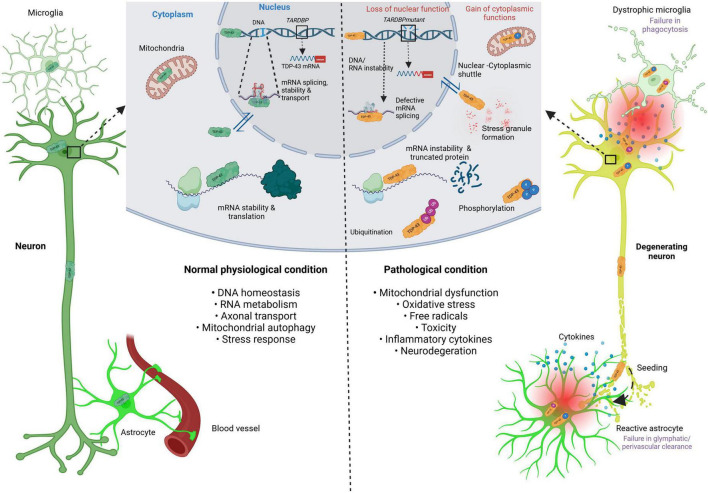
Schematic illustrating the molecular mechanism of TDP-43 formation and its signaling cascade involving inflammation and neurodegeneration. This schematic illustrates the molecular mechanisms of TDP-43 formation under normal physiological conditions (left-hand side), and under pathological conditions (right-hand side). The dark green neuron and TDP-43 indicate normal phenotypes. The orange TDP-43 indicates pathological protein, either in mutation, defective splicing, mRNA instability, stress granule formation, ubiquitination and phosphorylation. TDP-43, TAR DNA-binding protein of 43 kDa; *TARDBP*; TAR DNA-binding protein of 43 kDa; mRNA, messenger ribonucleic acid; DNA, deoxyribonucleic acid; RNA, ribonucleic acid. Created in BioRender https://BioRender.com/d68v242.

A hallmark of many neurodegenerative diseases (NDDs) is the abnormal misfolding, aggregation, and accumulation of various proteins in neuronal cells. NDDs characterized by pathological cytoplasmic inclusions of TDP-43 are collectively termed TDP-43 proteinopathies (Liao et al., 2022). The loss of TDP-43’s normal function in the nucleus, combined with its toxic gain of function in the cytoplasm, may contribute to the mechanisms underlying TDP-43 proteinopathy (Scotter et al., 2015; Arseni et al., 2022). Notably, its pathogenicity depends on its mislocalization and aggregation in the cytoplasm, where it is phosphorylated and ubiquitinated, leading to neurotoxicity (Miguel et al., 2011; Tamaki and Urushitani, 2022; de Boer et al., 2021). TDP-43 aggregates are also thought to have a prion-like quality and seeding propensity, which can aggravate its accumulation (Smethurst et al., 2016; Cordts et al., 2023). Figure 1 is a schematic illustrating the molecular mechanisms of TDP-43 formation and its signaling cascade involving inflammation and neurodegeneration.

### 1.1 Overview of TDP-43 proteinopathies

TDP-43 aggregates have been identified in the central nervous system (CNS) of 97% of amyotrophic lateral sclerosis (ALS), 45% of frontotemporal lobar degeneration (FTLD, termed FTLD-TDP), 19–57% of Alzheimer’s disease (AD), as well as in numerous other NDDs, with distribution patterns corresponding to characteristic stages within these diseases (Tamaki and Urushitani, 2022; Cordts et al., 2023; Meneses et al., 2021; Liao et al., 2022).

#### 1.1.1 ALS

ALS is a rare, aggressive disease of the upper and/or lower motor neurons, leading to the eventual loss of voluntary muscle function due to progressive neurodegeneration (Rojas et al., 2020). The peak incidence of ALS occurs between the ages of 70–79, with the age of onset being 58–63 years for sporadic ALS (sALS) and 40–60 years for familial ALS (fALS) (Ingre et al., 2015). About 10% of ALS cases are inherited becoming fALS while the other 90% occur sporadically becoming sALS (Xu and Yang, 2014). TDP-43 aggregate pathology is seen in 97% of ALS cases (Scotter et al., 2015; Tamaki and Urushitani, 2022). TDP-43 aggregates in ALS initially start in the agranular motor cortex, brainstem motor nuclei, and spinal motor neurons, and then spread to the nearby frontal, temporal, and parietal lobes (Liao et al., 2022; Arseni et al., 2022). Skein-like inclusions, which are tubular, thread-like structures composed of primarily aggregated and ubiquitinated TDP-43 protein, are characteristic of ALS (Arai et al., 2006; Neumann et al., 2006).

In fALS, mutations in chromosome 9 open reading frame 72 (*C9orf72*), *TARDBP*, RNA-binding protein fused in sarcoma (*FUS)*, and superoxide dismutase (*SOD1*) collectively account for approximately 70% of cases and are associated with toxic effects (Baldacci et al., 2020; Scotter et al., 2015). Mutant *C9orf72* is thought to upregulate TDP-43 in the retina by promoting RNA dysregulation (Estes et al., 2013) and mislocalization into the cytoplasm (Cook et al., 2020). *FUS* is thought to upregulate TDP-43 in a comparable way via dysregulation of its transcript (Wang et al., 2011), promoting mislocalization (Ederle et al., 2018), and aggregation (Hergesheimer et al., 2019). *SOD1* upregulates TDP-43 accumulation by increasing mislocalization (Zeineddine et al., 2017) and aggregation (Hergesheimer et al., 2019). Patients with *TARDBP* will invariably exhibit TDP-43 pathology since it codes for TDP-43 (Xu and Yang, 2014). ALS caused by mutations in *SOD1* and *FUS* does not exhibit TDP-43 pathology, suggesting a distinct pathogenic mechanism (Scotter et al., 2015). Conversely, certain ALS-related mutations can increase the aggregation propensity and cytotoxicity of TDP-43, potentially leading to toxic gain-of-function (de Boer et al., 2021). Cognitive deficits occur in 10–15% of ALS cases (Baldacci et al., 2020), and it is well-established that ALS closely overlaps biologically and clinically with frontotemporal dementia (FTD). FTD is a clinical syndrome characterized by progressive changes in personality, behavior, and/or language. FTLD refers to the degeneration of the frontal and temporal lobes, with FTD being one presentation of FTLD. (Babazadeh et al., 2023; Dijkstra et al., 2023; Mackenzie and Neumann, 2016; Arseni et al., 2022).

#### 1.1.2 FTD

FTD is the second most common presenile or early-onset dementia after AD and has many variants, including behavioral, language, and/or motor dysfunction (Arseni et al., 2022; Dijkstra et al., 2023), with one of the three main pathological subtypes being TDP-43 proteinopathy (Baldacci et al., 2020). A family history is present in 40% of FTD cases, with the most common genetic causes being *C9orf72* expansion, and mutations in microtubule-associated protein tau (*MAPT*) and granulin precursor (*GRN*) (Baldacci et al., 2020). The average age of symptom onset is 49.5, 58.2, and 61.3 years for the *MAPT*, *C9orf72*, and *GRN* variants, respectively, according to a large-scale retrospective study by Moore et al. (2020). The spreading pattern of TDP-43 proteinopathy of FTD is thought to be like that of ALS. The behavioral variant of FTD (bvFTD) includes symptoms such as disinhibition, apathy, loss of sympathy/empathy, compulsive behavior, hyperorality, with relative sparing of memory and visuospatial functions. Additionally, bvFTD exhibits the most extensive TDP-43 pathology, developing extensive orbitofrontal cortical pathology that eventually spreads to the spinal cord motor neurons and occipital areas (Baldacci et al., 2020; Brettschneider et al., 2014; Liao et al., 2022; Cordts et al., 2023).

#### 1.1.3 AD

AD is the primary cause of dementia in the elderly, characterized by early impairment of episodic memory and gradual cognitive decline (Baldacci et al., 2020; Liao et al., 2022). The incidence and prevalence increase with age; however, 5% of patients present with early-onset AD before the age of 65 (Awada, 2015). Patients with TDP-43 co-pathology (∼19–57%) tend to have worse cognitive impairment and outcomes (Tamaki and Urushitani, 2022; Meneses et al., 2021; Liao et al., 2022). TDP-43 pathology is more common in individuals with a common genetic risk factor, apolipoprotein ε4 allele (*APOE4*) (Meneses et al., 2021). In AD, TDP-43 pathology is most frequently seen in limbic regions, often co-localizing with neurofibrillary tangles and alpha-synuclein (Meneses et al., 2021; Baldacci et al., 2020). However, in later stages, it may spread to the temporal and frontal cortices (Liao et al., 2022). Conversely, limbic-predominant age-related TDP-43 encephalopathy (LATE) is an amnestic dementia usually affecting older adults (25–50% being older than 80 years), where TDP-43 neuropathology is localized to the hippocampus and amygdala, spreading to the middle frontal gyrus in later stages (Cordts et al., 2023; de Boer et al., 2021; Liao et al., 2022). AD and LATE are genetically pleiotropic, with the *APOE4* risk allele associated with both conditions. Further, these conditions are often found together in the same brains, referred to as AD-LATE, and mixed pathology is associated with more rapid cognitive decline (Nelson et al., 2022).

#### 1.1.4 CTE

Chronic traumatic encephalopathy (CTE) results from repetitive mild traumatic brain injuries or concussions (McKee et al., 2013; Phansalkar et al., 2023). While predominantly a tauopathy, approximately 85% of cases develop TDP-43 proteinopathy (McKee et al., 2013; de Boer et al., 2021). Males with a history of contact sports are the demographic most likely to develop CTE (McKee et al., 2013; Phansalkar et al., 2023). The average age of onset for neuropathologically confirmed CTE is 44.3 years, with a wide range of spanning 16–83 years (Kriegel et al., 2018). Stages I-III involve more focal distribution patterns of hyperphosphorylated tau, whereas Stage IV refers to widespread tauopathy (McKee et al., 2013). TDP-43 pathology tends to appear in the frontal cortex, with or without a limbic distribution (Nicks et al., 2023). The presence of TDP-43 proteinopathy in CTE confirms that an underlying genetic cause is not always necessary, as is often the case in the ALS-FTD spectrum.

Overall, NDDs are a group of acquired, debilitating conditions characterized by the progressive dysfunction and death of neurons in the brain and nervous system (Davenport et al., 2023). They can severely impact the quality of life by causing gradual declines in cognition and memory, mood and sleep disturbances, motor impairments, and death (Chalkias et al., 2021; Morrone et al., 2023; Babazadeh et al., 2023). While treatments for some of these diseases are available to manage symptoms and slow progression, they remain incurable (Davenport et al., 2023). NDDs such as ALS, FTLD, AD, and CTE are categorized individually but are understood to exist on a spectrum with overlapping pathogenic mechanisms and phenotypes (Baldacci et al., 2020; Nelson et al., 2022; Fawzi et al., 2014; McKee et al., 2013). Furthermore, many patients exhibit disease heterogeneity and comorbidities, complicating diagnosis. Due to overlapping phenotypes, symptom heterogeneity, the topographic distribution of TDP-43 neuropathology, and the often insidious nature of NDD development, these diseases are usually definitively confirmed via neuropathological examination of brain lesions upon autopsy, resulting in delays in diagnosis (Baldacci et al., 2020; Cordts et al., 2023). There is a clear need for accurate detection of NDDs, particularly in their early, pre-clinical stages, to stratify patients and expand the treatment window. With recent advancements and increased focus on TDP-43 proteinopathies, recognizing TDP-43 disease pathology as an underlying molecular mechanism holds promise as a potential biomarker.

### 1.2 TDP-43 as a potential biomarker for NDDs

There is growing evidence that TDP-43 proteinopathy extends beyond the brain. Recent advancements have explored the use of TDP-43 as a biomarker for TDP-43 proteinopathies in various biofluids, such as serum, cerebrospinal fluid, and tear film (Cordts et al., 2023; Chalkias et al., 2021; Majumder et al., 2018; Giampietri et al., 2022). However, detection has been challenging because most antibodies bind the pathological and physiological forms of TDP-43 (Cordts et al., 2023). Furthermore, although some of these biofluids have a direct connection to the brain, they do not share the same embryological origin making direct comparisons with neuropathology difficult.

On the other hand, the eye has been considered a “window” to the brain (Vautier et al., 2023; Dijkstra et al., 2023). The retina is of particular interest due to its direct connection to the brain via the optic nerve and shared embryological origins (Chalkias et al., 2021). The retina in subjects with NDDs has been studied using optical coherence tomography (OCT), revealing findings such as retinal and macular thinning, changes in vasculature, or axonal degeneration in ALS, FTD, and AD (Soldatov et al., 2021; Vautier et al., 2023; Wong et al., 2022; Kim et al., 2017; Chalkias et al., 2021). However, these findings can also be found in non-neurological diseases such as obesity (Christinaki et al., 2022; González-Martín-Moro et al., 2023) and chronic kidney disease (Laiginhas et al., 2019; Paterson et al., 2020), as well as in response to extrinsic factors like air pollution and oral contraceptive use (Chua et al., 2020; Shaaban and Badran, 2019). Thus, there is potential value in directly measuring pathological TDP-43 in the eye, as it reflects the disease process and has a direct connection to the brain. This narrative review explores the role of TDP-43 proteinopathy in various NDDs, with a focus on its potential as a biomarker in the eye, particularly the retina, for early detection and diagnosis.

### 1.3 TDP-43 in the eye using animal models

Research on TDP-43 using animal models has significantly advanced our understanding of its role in neurodegenerative diseases. Here, the focus will be on the effects of pathological TDP-43 in retinal degeneration.

The effect of TDP-43 mislocalization and aggregation in the cytoplasm has yielded mixed findings. It has been shown in *Drosophila melanogaster* that cytoplasmic accumulation of TDP-43 is toxic to the cells of the developing eyes, including photoreceptor neurons (Miguel et al., 2011), and in the retinal ganglion cell layer (RGC) and the optic nerve (Atkinson et al., 2021). Zhang et al. (2021) highlight the role of TDP-43 cytoplasmic aggregation in RGC apoptosis and decreased retinal thickness in a glaucoma-associated (E50K optineurin) mouse model in the context of ALS. Further investigations of fALS-linked-mutant of Profilin 1 (PFN1) showed increased TDP-43 mislocalization followed by retinal degeneration in *D. melanogaster*, as shown by Matsukawa et al. (2016), while co-expression of mutant PFN1 with wild type (WT) PFNI did not result in retinal degeneration. However, Ward et al. (2014) found with progranulin (*GRN*) deficiency, a significant cause of familial FTLD-TDP, retinal neuron loss occurs independently of cytoplasmic TDP-43 aggregation in GRN-KO mice because of reduced GTPase Ran expression. Lanson et al. (2011) found that WT FUS/TLS and mutant TDP-43 (M337V) or mutant FUS/TLS (R521H) and WT TDP-43 in flies are combinations with synergistic effects of eye degeneration. Further, Cragnaz et al. (2014) proposed that the cytoplasmic aggregation of TDP-43 (TBPH in *D. melanogaster*) might have a protective role against ocular degeneration, suggesting a complex relationship between protein aggregation and neurotoxicity. These indicate a need for further investigations into the extent to which TDP-43 mislocalization has on retinal degeneration.

Sporadic ALS and fALS-related TDP-43 mutants (i.e., A315T, D169G, G298S, and N345K) have been found to cause retinal degeneration in *D. melanogaster* to variable degrees (Estes et al., 2011; Estes et al., 2013; Ihara et al., 2013). Further, overexpression of WT TDP-43 itself causes retinal degeneration (Atkinson et al., 2021; Ihara et al., 2013), and even more so compared to the sALS and fALS mutants (Estes et al., 2011). Some mutants have a less severe toxic effect compared to others, like D169G because of its location in the RNA binding domain RRM1 which is thought to be crucial for mediating neurotoxicity (Estes et al., 2013). However, future research is needed to clarify why certain TDP-43 mutants cause lesser or greater retinal degeneration compared to others. Overall, different TDP-43 proteinopathy-related mutants lead to varied pathological outcomes, suggesting that personalized approaches may be necessary for treating TDP-43 proteinopathies.

The interactions of TDP-43 with other proteins have also been a focus of research. Uechi et al. (2018) investigated ubiquitin-binding protein CG5445 in *D. melanogaster* and suggested that it enhances the solubility and proteasomal degradation of mutant TDP-43 aggregates, rescuing eye degeneration. Similarly, Ambegaokar and Jackson (2011) found that in flies a reduced dosage of glycogen synthase kinase-3b (GSK-3b) can suppress TDP-43 toxicity, and that its activity is strongly increased when there is mutant TDP-43^*G*331*K*^ (linked to ALS) expression. Appocher et al. (2017) examined key heterogeneous nuclear ribonucleoprotein (i.e., Hrb27c, CG42458, Glo, and Syp) proteins and found that their silencing strongly decreased TDP-43 (TBPH in flies) gain-of-function toxicity in *D. melanogaster* eyes, making them significant suppressors. Importantly, DAZAP1 (the human homolog of Hrb27c) was shown to correct pre-mRNA splicing events altered by TDP-43 depletion in human neuronal cell lines. Meanwhile, Crippa et al. (2016) discovered that overexpression of heat shock protein (HSP) 67Bc, can suppress eye degeneration caused by cytoplasmic accumulation of a TDP-43 in *D. melanogaster*. Kim et al. (2014) showed that inhibiting eIF2α phosphorylation, indicative of stress granule formation, mitigates TDP-43 toxicity in both *D. melanogaster* and mammalian neurons (derived from Long Evans rat embryo). Pons et al. (2017) looked at *D. melanogaster* eyes and found that downregulation of splicing factor 3b subunit 1 (Sf3b1) or expression of RNA-binding protein 1 (Rbp1) proteins resulted in increased TDP-43 protein steady-state level. Expression of serine-arginine-rich splicing factor 2 (SF2) resulted in TDP-43 accumulation with its knock-down producing the opposite effect. These findings highlight the various protein interactions with TDP-43 that mitigate retinal degeneration. Expanding research on such proteins can unveil potential therapeutic targets for TDP-43 proteinopathies.

**FIGURE 2:**
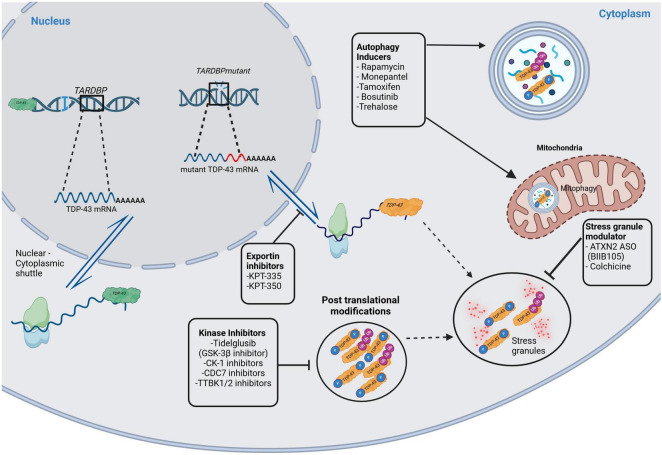
Therapeutic targets in the TDP-43 signaling pathways. This figure illustrates potential therapeutic targets in TDP-43 signaling pathways. The green colored TDP-43 indicate physiologically normal protein, whereas the orange colored TDP-43 are pathological protein. Kinase inhibitors (of GSK-3b, CK-1, CDC-7, TTBK1/2) inhibit post-translational modifications like phosphorylation and ubiquitination of TDP-43. Exportin inhibitors (KPT-335, KPT-350) decrease TDP-43 mislocalization in the cytoplasm. There are also stress granule modulators that act on ATXN2 ASO and colchicine. Finally, there are autophagy inducers like Rapamycin, Monepantel, Tamoxifen, Bosutinib, and Trehalose. GSK-3b, glycogen synthase kinase 3b; CK-1, casein kinase 1; CDC7, cell division cycle 7 kinase; TTBK1/2, tubulin and tau kinase 1 and 2; KPT-335 and KPT-350, exportin inhibitors; ATXN2 ASO, ataxin-2 antisense oligonucleotide. Created in BioRender https://BioRender.com/d68v242.

Although not explicitly studied in the retina, several other therapeutic targets for TDP-43 proteinopathy have potential. Inhibitors of GSK-3b, casein kinase 1 (CK-1), cell division cycle 7 kinase (CDC-7), and tau and tubulin kinase 1/2 (TTBK1/2) have been found to reduce post-translational modifications like phosphorylation and ubiquitination (Hayes and Kalab, 2022; Palomo et al., 2019). Exportin inhibitors of KPT-335 and KPT-350 have shown promise in limiting TDP-43 mislocalization to the cytoplasm (Hayes and Kalab, 2022; Palomo et al., 2019). For autophagy induction, Rapamycin, Monepantel, Tamoxifen, Bosutinib, and Trehalose have shown promise in clearing TDP-43 aggregates (Babazadeh et al., 2023; Hayes and Kalab, 2022; Palomo et al., 2019). Finally, stress granule modulators like ataxin-2 antisense oligonucleotide (ATXN2 ASO) and Colchicine may inhibit the formation of granules containing phosphorylated and ubiquitinated TDP-43 (Babazadeh et al., 2023; Hayes and Kalab, 2022). Figure 2 illustrates these therapeutic targets in the TDP-43 signaling pathways.

**FIGURE 3:**
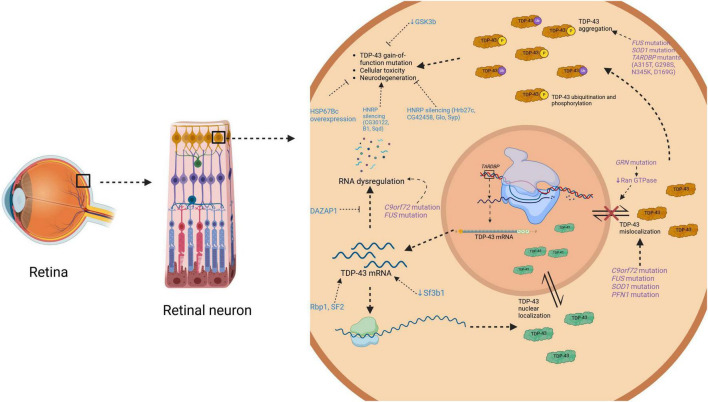
Diagrammatic representation on how toxic mutant proteins found in neurodegenerative disease and in animal models cause TDP-43 upregulation in the retina. This schematic illustrates an overview of where certain mutations like *C9orf72, SOD1, TARDBP, FUS, PFN1*, and *GRN* have toxic effects in TDP-43 molecular pathways causing upregulation. It also illustrates where various proteins (i.e., GSK-3b, hnRPs, HSP67Bc, etc.) studied in animal models of TDP-43 in the eye have their toxic effects. TDP-43, TAR DNA-binding protein of 43 kDa; mRNA, messenger ribonucleic acid; *C9ord72*, chromosome 9 open reading frame 72; *FUS*, fused in sarcoma; *SOD1*, superoxide dismutase; *PFN1*, profilin 1; *TARDBP*; TAR DNA-binding protein of 43 kDa; *GRN*, granulin precursor; GSK3b; glycogen synthase kinase-3b; HNRP, heterogeneous nuclear ribonucleoprotein; HSP67Bc, heat shock protein 67Bc; DAZAP1, DAZ associated protein 1; Rbp1, RNA-binding protein 1; Sf3b1, splicing factor 3b subunit; SF2, serine-arginine-rich splicing factor 2. Created in BioRender https://BioRender.com/a82z379.

Overall, there are mixed results regarding the pathogenicity of TDP-43 mislocalization and various TDP-43 proteinopathy-related mutants, as well as potential therapeutic targets. However, TDP-43 accumulations in the eye indicate NDD pathology, and expanded research into its pathogenic molecular pathways furthers its potential as a retinal biomarker for NDDs in humans. A detailed summary of this information can be found in Table 1. Figure 3 is a schematic representing how toxic NDD-related mutant proteins and other various proteins (i.e., heat shock proteins, splicing factors, ubiquitin binding proteins, etc.) studied in the animal models discussed cause upregulation of TDP-43 in the retina.

**TABLE 1:** Summary of animal model studies investigating pathological TDP-43 in the eye.

No.	References	Animal model	Objectives	Key findings
1	Ambegaokar and Jackson (2011)	*Drosophila melanogaster*	To investigate modifiers of tauopathy via functional genomic screening.	Reduced dosage of GSK-3b can suppress TDP-43 toxicity, and its activity is strongly increased when there is mutant TDP-43^G331K^ (linked to ALS) expression.
2	Appocher et al. (2017)	*D. melanogaster*	To test how major hnRNPs affect TDP-43 overexpression and deletion in fly eyes.	Strong suppressors: Hrb27c, CG42458, Glo, Syp, Hrp38 Mild suppressors: Rump, Heph, Sm, Hrb87F Enhancers: CG30122, B1, Sqd
3	Atkinson et al. (2021)	Mouse (C57BL/6J)	To investigate the impact of TDP-43 mislocalization on neuronal cell bodies.	Both hTDP-WT-GFP and hTDP-ΔNLS-GFP mutant TDP-43 mislocalization leads to increased retinal neurofilament heavy expression and decreased numbers of neurofilament-positive axons.
4	Cragnaz et al. (2014)	*D. melanogaster*	To investigate the effects of a cellular model of TDP-43 Q/N rich amino acid sequence 331-369 repeated 12 times (12xQ/N) *in vivo* on eye development.	Induced aggregation of TDP-43 has a protective role on fly eye development.
5	Estes et al. (2011)	*D. melanogaster*	To study the effects of the A315T mutant of TDP-43 in comparison to WT overexpression.	Both A315T allele and WT TDP-43 had toxic effects on the retina in a dose-dependent manner.
6	Estes et al. (2013)	*D. melanogaster*	To investigate the effects of additional TDP-43 mutants (i.e., D169G, G298S, and N345K).	Overexpression of the TDP-43 mutants leads to age- and dose-dependent neurodegeneration in fly neuroepithelium.
7	Ihara et al. (2013)	*D. melanogaster*	To investigate how fALS-linked TDP-43 mutations impact neurotoxicity when compared to NLS mutant and WT.	Overexpression of WT TDP-43 resulted in retinal degeneration and was exacerbated by fALS-linked mutations (G2985, A315T, M337V, Q343R) or disruption of NLS. Further, abolishing TDP-43’s RNA binding capacity mitigated neurodegeneration.
8	Kim et al. (2014)	*D. melanogaster*, Long Evans rat embryo, yeast	To identify modifiers of disease genes implicated in TDP-43 toxicity relevant to human disease.	Inhibition of eIF2alpha phosphorylation, which is indicative of stress granule formation, TDP-43 toxicity in both D. melanogaster and mammalian neurons is mitigated.
9	Lanson et al. (2011)	*D. melanogaster*	To investigate the genetic interactions between FUS and TDP-43 in FUS-related neurodegeneration.	Lanson and colleagues (2011) found that WT FUS/TLS and mutant TDP-43 (M337V) or mutant FUS/TLS (R521H) and WT TDP-43 in flies are combinations with synergistic effects of eye degeneration.
10	Matsukawa et al. (2016)	*D. melanogaster*	To elucidate the mechanism whereby fALS-linked *PFN1* mutation led to neuronal death.	Double overexpression of TDP-43 and mutant PFN1 exacerbated retinal degeneration, led to mislocalization of TDP-43 and increased neurodegeneration, while co-expression with WT PFN1 did not result in similar retinal degeneration.
11	Miguel et al. (2011)	*D. melanogaster*	To address the mechanism underlying TDP-43 toxicity *in vivo* using two mutant forms of TDP-43; hTDP-43mutNLS and hTDP43mutNES.	Only cytoplasmic accumulation of TDP-43 were toxic to retinal cells, while both cytoplasmic and nuclear accumulations were toxic to neurons of the brain.
12	Pons et al. (2017)	*D. melanogaster*	To investigate how splicing factors modulate TDP-43 production.	Downregulation of Sf3b1 or expression of Rbp1 proteins resulted in increased TDP-43 protein steady-state level. Expression of SF2 resulted in TDP-43 accumulation with its knock-down producing the opposite effect.
12	Uechi et al. (2018)	*D. melanogaster*	To explore how ubiquitin-binding protein CG5445 affects aggregation of TDP-43 in fly eyes.	CG5445 enhances the solubility and proteasomal degradation of mutant TDP-43 aggregates, indicating a potential therapeutic target for ALS.
13	Ward et al. (2014)	Mouse (GRN-KO)	To investigate how the lack of GRN, a common cause of familial FTLD-TDP, impacts neurodegeneration in mice.	Without GRN, there is nuclear depletion of TDP-43 in the RGC layer, followed by retinal neuron loss, suggesting neurodegeneration may occur independently of cytoplasmic TDP-43 aggregation.
14	Zhang et al. (2021)	Mouse	To investigate whether the glaucoma-associated OPTN (E50K) mutation causes TDP-43 aggregation via impaired autophagic degradation.	E50K OPTN mutation was linked to impaired autophagic flux of TDP-43, leading to RGC apoptosis and decreased retinal thickness.

TDP-43, TAR DNA binding protein of 43 kDa; hnRNP, heterogeneous nuclear ribonucleoprotein protein; ERG, electroretinogram; ALS/FTD, amyotrophic lateral sclerosis/frontotemporal lobar dementia; fALS, familial amyotrophic lateral sclerosis; FXTAS, Fragile X tremor ataxia syndrome; WT, wild-type; NLS, nuclear localization sequence; PFN1, profilin 1; GRN, progranulin; RGC, retinal ganglion cell; OPTN, optineurin; GRN-KO, progranulin knock-out; MAPK, mitogen-activated protein kinase; Sf3b1, splicing factor 3b subunit 1; Rbp1, RNA-binding protein 1; SF2, serine-arginine-rich splicing factor 2; FUS/TLS, fused in sarcoma/translated in liposarcoma; GSK-3b, glycogen synthase kinase-3b.

### 1.4 TDP-43 as a potential retinal biomarker for NDDs in humans

#### 1.4.1 Retinal TDP-43 pathology in the ALS-FTD spectrum

Various research groups have utilized immunohistochemistry to investigate potential retinal TDP-43 pathology in NDDs. Dijkstra et al. (2023) found pTDP-43 positive inclusions within the outer plexiform layer (OPL) of the retina in FTLD-TDP subjects with *C9orf72* expansion (*n* = 4), progranulin (*PRGN)* (*n* = 1), sporadic type E (*n* = 1), and sporadic type C (*n* = 1) mutations. However, they did not find TDP-43 positive inclusions in cases of other FTD variants or ALS-TDP. Although cortical TDP-43 pathology was not shown, the authors postulated that donors with the most extensive cortical and subcortical pathology exhibited the greatest retinal TDP-43 pathology. They introduced the idea that retinal TDP-43 pathology might correlate with brain TDP-43 pathology.

Similarly, Fawzi et al. (2014) found that in an ALS case with a *C9orf72* expansion mutation, TDP-43 retinal pathology was absent; however, they did find p62 and ubiquitin-positive cytoplasmic inclusions, primarily (94.9%) in the inner nuclear layer (INL), likely within amacrine and horizontal cells (Fawzi et al., 2014; Volpe et al., 2015). Additionally, TDP-43 positive skein-like inclusions were detected in the hypoglossal nucleus of the brainstem and in lumbar, cervical, and thoracic spinal motor neurons. This finding suggests that the subject had at least Stage I ALS (Liao et al., 2022), which may indicate a minimum burden of brain TDP-43 pathology required to see retinal pathology. The same group assessed another subject with ALS and a 12-year history of FTD. They found no retinal aggregates, nor was cortical pathology assessed (Volpe et al., 2015).

On the other hand, Pediconi et al. (2023) demonstrated that in 10 cases of sALS (mutations not specified), there were TDP-43 and p62 positive cytoplasmic inclusions in the ganglion cells and the INL. However, potential TDP-43 pathology in the CNS was not assessed, nor was ALS staging provided. While these authors have observed the presence of TDP-43 in the retina, its potential utility as a biomarker is limited without a corresponding evaluation of the TDP-43 pathology burden in the brain to confirm the underlying neuropathology.

Hart de Ruyter et al. (2024) investigated the presence of hallmark proteins in various NDDs, including FTLD-TDP, AD, tauopathies, synucleinopathies, etc. They compared immunohistochemistry findings in the post-mortem retina to those in the brain. Typical neuronal cytoplasmic inclusions of pTDP-43 were found in the INL, and short dystrophic neurites were observed in the OPL in 7 out of 8 cases of FTLD-TDP. In 1 out of 8 cases of FTLD-TDP, there was brain pathology, but no TDP-43 was detected in the retina. They also found inclusions related to AD-related pathologies, which will be discussed in section 1.4.2.

#### 1.4.2 Retinal TDP-43 pathology in the AD-LATE spectrum

Williams et al. (2017) performed staining for multiple pathologies including tau, β-amyloid, and TDP-43, on 11 cases of AD at various stages, none of which stained positively for TDP-43 in the retina. Additionally, they assessed potential co-existing eye pathologies, such as glaucoma and macular degeneration (MD). This examination allowed the authors to determine whether the findings were specific to AD or could be confounded by eye diseases that affect similar structures, such as the retina and optic nerve. They concluded that protein deposits in the eye do not play a prominent role in the pathogenesis of MD or glaucoma in patients with AD. The authors further analyzed the brains of the pathology cases, finding that only one had potential TDP-43 proteinopathy in the form of hippocampal sclerosis with TDP-43 positive inclusions. This suggests that the severity of brain pathology may not correlate with eye pathology.

Hart de Ruyter et al. (2024) found pTDP-43 retinal inclusions in typical AD (3/10), atypical AD (1/3) and synucleinopathies (5/22). In one case of atypical AD and synucleinopathy, there was no corresponding brain TDP-43 pathology. Regarding LATE staging, 21 cases were reported to have LATE, and the likelihood of finding retinal pTDP-43 increased with LATE stages 2 (OR = 14.59, *P* = 0.002) and 3 (OR = 13.10, *P* < 0.03). They found that, in general, the retina primarily manifested protein aggregates associated with the primary NDD (Hart de Ruyter et al., 2024). Among all the proteins studied, TDP-43 was the least prevalent in both the retina and brain. However, these mixed findings may support the idea that brain pathology may not always correlate with ocular pathology (Williams et al., 2017).

#### 1.4.3 Retinal TDP-43 pathology in CTE

**FIGURE 4:**
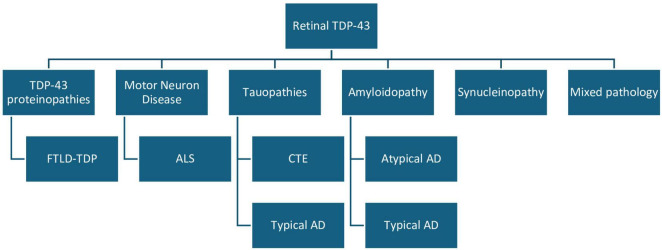
Summary of neurodegenerative diseases that were positive for TDP-43 in human post-mortem retina specimens. This categorically displays the NDDs that tested positively for pathological TDP-43 in human specimens using immunohistochemistry. TDP-43, TAR DNA-binding protein of 43 kDa; FTLD, frontotemporal lobar degeneration; ALS, amyotrophic lateral sclerosis; AD, Alzheimer’s disease; CTE, chronic traumatic encephalopathy; NDD, neurodegenerative disease.

Phansalkar et al. (2023) investigated potential TDP-43 retinal pathology in individuals with stage IV CTE, all of whom had histories of contact sports. The sample size was small (*n* = 8), however, they observed strong pTDP-43 cytoplasmic inclusions in six cases. The scattered punctate inclusions were found in the INL of retinal laminar cells, most likely in horizontal cells. Of the 8 CTE retinas, 6 showed variable degrees of pTDP-43 positive inclusions in the spinal cord, amygdala, hippocampus, entorhinal cortex, inferior temporal cortex, and neocortex. One of the cases with negative TDP-43 retinal pathology did, however, show cortical involvement. There was limited ophthalmic history for all subjects; however, none, showed signs of atrophy. Another limitation of this study was that among the controls, most had neuropathologic changes, including two with mild to moderate Alzheimer’s disease changes. This, coupled with the incomplete cortical assessment for TDP-43, makes it challenging to determine to what extent TDP-43 cortical and retinal burden may correlate. Given these results, it remains unclear whether a CTE stage less than IV would yield retinal TDP-43 pathology. Figure 4 summarizes the NDDs that were positive for TDP-43 in human port-mortem specimens. Table 2 summarizes the findings in human subjects discussed in sections 1.4.1–1.4.3.

**TABLE 2:** Summary of studies testing for pathological TDP-43 aggregates in the human post-mortem retina.

No.	References	Analytical method	Sample size (*n*)	TDP-43 status in CNS	TDP-43 status in retina	Remarks
1	Dijkstra et al. (2023)	Immunohistochemistry and double immuno-fluorescence	Total: 7 FTLD-TDP-A (*PGRN*): 1 FTLD-TDP-B (*C9orf72*): 4 FTLD-TDP-C (sporadic): 1 FTLD-TDP-E (sporadic): 1	Not specified	OPL	TDP-43 inclusions do not colocalize with horizontal or amacrine cells.
			Total: 17 Control: 6 FTD-FUS: 1 FTD-TAU-MAPT: 1 CBD: 2 PSP: 2 ALS-TDP: 2 AD; no TDP: 1 AD; limbic TDP: 2		Negative	
2	Fawzi et al. (2014)	Immunohistochemistry	ALS (*C9orf72*): 1	Positive; TDP-43 inclusions in the hypoglossal nucleus > lumbar anterior horn > cervical and thoracic anterior horns.	Negative	TDP-43 inclusions in CNS were noted as “skein-like.”
3	Hart de Ruyter et al. (2024)	Immunohistochemistry	Total: 17 Control (+): 1 FTLD-TDP: 7 Mixed pathology: 2 Synucleinopathy: 4 Typical AD: 3	Positive in the brain; location not specified.	INL and in short dystrophic neurites in the OPL.	
			Total: 4 Control (-): 1 Control (+): 1 Atypical AD: 1 Synucleinopathy: 1	Negative		
			Total: 13 FTLD-TDP: 1 Mixed pathology: 3 Primary tauopathy: 1 OND: 2 Synculeinopathy: 3 Typical AD: 3	Positive in the brain; location not specified.	Negative	
			Total: 67 Control (-): 6 Control (+): 15 Atypical AD: 3 Mixed pathology: 6 OND: 14 Primary tauopathy: 7 Synucleinopathy: 19 Typical AD: 7	Negative	Negative	
4	Volpe et al. (2015)	Immunohistochemistry	ALS with FTD: 1	Not specified	Negative	
5	Pediconi et al. (2023)	Immunofluorescence	Total: 21 Control: 11 ALS (sporadic): 10	Not assessed	Ganglion cells and INL.	Both cytoplasmic TDP-43 and pTDP-43 were higher in ALS. Presence of retinal cell degeneration in ALS.
6	Phansalkar et al. (2023)	Histology and immunohistochemistry	CTE (stage IV): 7	Positive; inclusions in the spinal cord, amygdala, hippocampus, and/or cortex.	INL retinal laminal cells	The TDP-43 inclusions are possibly within retinal horizontal cells. No sign of overt retinal degeneration
			Total: 9 Controls: 8 CTE (stage IV): 1	Negative for controls. Unknown for the CTE case.		
7	Williams et al. (2017)	Immunohistochemistry	Total: 17 Control: 2 AD: 16	Negative	Negative	Only 11 AD subjects were stained for pTDP-43.
			AD: 1	Positive; hippocampal sclerosis with TDP-43 inclusions.	Negative	

CNS, central nervous system; FTLD, frontotemporal lobar degeneration; FTD, frontotemporal dementia; FUS, fused-in-sarcoma; CBD, corticobasal degeneration; PSP, progressive supranuclear palsy; ALS, amyotrophic lateral sclerosis; AD, Alzheimer’s disease; CTE, chronic traumatic encephalopathy NC, neuropathologic change; TDP, TAR DNA binding protein; OPL, outer plexiform layer; INL, inner nuclear layer; OND, other neurodegenerative diseases; MAPT, microtubule-associated protein tau.

## 2 Discussion

Overall, studies investigating potential retinal TDP-43 pathology across various NDDs have produced mixed results. It remains unclear how strongly pathological retinal TDP-43 aggregation correlates to cortical burden in NDDs. However, several limitations exist in these studies. Firstly, most studies have small sample sizes, which introduces low statistical power, potential overestimation of effect sizes, low reproducibility, and limited generalizability. Secondly, not all studies employed well-established staging methods for disease severity, nor was brain pathology consistently determined for comparison. This is important because many NDDs involve abnormal accumulation and aggregation of misfolded proteins other than TDP-43, such as tau, β-amyloid, and α-synuclein, in both brain and eye tissues. In animal studies of TDP-43 proteinopathies, retinal dysfunction occurs before brain dysfunction (Gao et al., 2024; Ward et al., 2014); however, this finding could not be ascertained in human studies, as the burden of brain pathology was not consistently assessed. Thirdly, when examining retinal TDP-43 in post-mortem eyes, neither antemortem ophthalmologic pathologies nor detailed clinical histories were consistently recorded. Understanding antemortem eye pathology and the clinical history of the donor is crucial when measuring pathological TDP-43 in the eye, as it helps distinguish disease-specific pathology from other ocular conditions, correlate findings with visual symptoms, provide insights into disease progression, and account for potential confounding factors that may influence TDP-43 mislocalization and aggregation. The link between increased TDP-43 aggregation in the retina and brain of NDD patients in terms of clinical correlates is not well-understood. With respect to visual abnormalities that may occur because of TDP-43 aggregation, there is little to no correlation to cortical aggregate pathology as of now (Hart de Ruyter et al., 2024; Williams et al., 2017). Finally, many studies do not sufficiently compare retinal TDP-43 pathology across various NDDs simultaneously. This is essential for understanding disease specificity, clinicopathological correlations, disease progression, and shared pathogenic mechanism of TDP-43 proteinopathies. Future studies may consider following the methods of the recent work by Hart de Ruyter et al. (2024), wherein they had the largest sample size, a variety of pathologies, and a range of pathological proteins studied. Our findings suggest that pathological aggregates of TDP-43 in the human retina are most found in FTLD-TDP, ALS, and CTE, meaning these diseases may be the most feasible to accurately study the potential utility of TDP-43 as a retinal biomarker.

These studies highlight the presence of TDP-43 aggregate toxicity in both the brain and retina simultaneously, emphasizing the connection between these tissues. TDP-43 aggregates have been shown to have prion-like properties, allowing them to self-propagate among neighboring cells (Smethurst et al., 2016; Cordts et al., 2023). Previous works have shown that pathological TDP-43 aggregates can propagate from neurons to neurons (Jo et al., 2020), however, TDP-43 is expressed ubiquitously in many cell types (de Boer et al., 2021). More research is required to elucidate how TDP-43 seeds from cortical neurons, glial cells, and retinal neuronal cells. This mechanism could potentially explain the spread of pathology between the brain and retina and elucidate potential therapeutic targets.

Future directions may include developing retinal tracers that detect pathological TDP-43 aggregates in the retina in living subjects, such as AMDX2011P, which binds tau and TDP-43 (Babazadeh et al., 2023). Soloperto et al. (2022) have developed new fluorescent probes for detecting tau aggregates, which could potentially be adapted for TDP-43 detection. A novel BODIPY-based probe has shown promise in labeling hyperphosphorylated tau and oligomeric tau in a humanized cortical neuron model, opening up possibilities for cost-effective monitoring of protein aggregation states. This approach could be extended to develop similar probes for TDP-43 detection in the retina. Hyperspectral imaging of the retina, while not specifically tested on TDP-43, is being investigated as a potential biomarker for NDDs like AD (Hadoux et al., 2019). This technique could potentially be adapted to study TDP-43 pathology in the retina, given its ability to detect subtle changes in light scatter associated with protein aggregates. Although non-specific for TDP-43 proteinopathies, oculomics (which includes methods like OCT, ERG, and visual-evoked potentials) can allow for more comprehensive detection of structural changes in the retina (Suh et al., 2023). Quantitative detection methods for protein aggregates in the retina are advancing, with Irwin et al. (2016) successfully employing color deconvolution processes and object detection algorithms to quantify pathological burden in FTLD cases, including those with TDP-43 proteinopathy (Irwin et al., 2016). Leveraging artificial intelligence, these methods can be complemented by machine learning algorithms that detect pathological TDP-43 in the brain to more accurately classify and differentiate between various NDDs based on regional TDP-43 severity (Muñoz et al., 2024; Young et al., 2023).

## 3 Conclusion

In conclusion, while the retina holds promise as a potential biomarker for NDDs involving TDP-43 proteinopathy, current evidence is inconsistent and limited by various methodological limitations. However, our findings suggest that pathological aggregates of TDP-43 in the human retina are most found in FTLD-TDP, ALS, and CTE, meaning these diseases may be the most feasible to accurately study the potential utility of TDP-43 as a retinal biomarker. Animal studies have been valuable for elucidating TDP-43’s pathogenic mechanisms; the effects of various mutants, proteins, and potential therapeutic targets. Further research in humans with larger sample sizes, consistent brain pathology assessments, and detailed clinical information is needed to establish the utility and reliability of retinal TDP-43 pathology as a biomarker for NDDs. Additionally, comparisons across multiple NDDs are essential to understand the specificity of retinal TDP-43 pathology, disease progression, and the shared pathogenic mechanisms of TDP-43 proteinopathies. Future directions for the TDP-43 as a retinal biomarker for NDDs include retinal tracers, hyperspectral imaging, oculomics, and machine learning development.
